# BioDRUMs: An Open-Source Python Pipeline for Automated Mean Drug-to-Antibody Ratio and Biologics Structural Integrity Analysis from High-Resolution Mass Spectrometry Data

**DOI:** 10.1021/jasms.6c00289

**Published:** 2026-10-05

**Authors:** Andrea Di Ianni, Francesco Molinaro, Luca M. Barbero, Kyra Cowan, Federico Riccardi Sirtori

**Affiliations:** † NBE-DMPK Innovative BioAnalytics, 26200 Merck Serono RBM S.p.A., an Affiliate of Merck KGaA, Darmstadt, Germany, via Ribes 1, Colleretto Giacosa, Turin 10010, Italy; ‡ University of Turin, Molecular Biotechnology Center, Department of Molecular Biotechnology and Health Sciences, University of Turin, Turin 10126, Italy; § New Biological Entities, Drug Metabolism and Pharmacokinetics (NBE-DMPK), Research and Development, Merck KGaA, Frankfurterstrasse 250, Darmstadt 64293, Germany

**Keywords:** drug-to-antibody ratio, data analysis automation, antibody-drug conjugates, biologics, intact mass analysis, biotherapeutics development

## Abstract

Drug-to-antibody ratio and structural integrity represent two critical quality attributes for antibody-drug conjugates and multispecific biologics in development. Intact mass spectrometry analysis of biologics is crucial to understanding the behavior of a new candidate, especially for complex modalities. Sample preparation is relatively robust and easy to reproduce in different laboratories and by different operators. However, mass spectrometric analysis and data processing represent the two most limiting steps. So far, no automated open-source pipeline has been published, which can automatically postprocess deconvoluted spectrum outputs from different vendors, plot data, and provide an overall interpretation of mean DAR and deep ADC characterization, especially on *in vivo* samples. BioDRUMs offers different features that complement existing commercial mass spectrometry deconvolution tools, such as customizable degradation-hypothesis generation, isobaric-species ambiguity reporting, vendor-neutral postprocessing of deconvoluted mass tables, structural-integrity scoring, automated plotting, and reporting. Streamlining data analysis of the mean drug-to-antibody ratio and structural integrity can accelerate and provide uniform data outputs and result delivery across different *in vitro*/*in vivo* studies. Moreover, a user-friendly pipeline would enable scientists to perform such an analysis for the first time more confidently, without requiring any pre-existing coding skills. In this work, we present BioDRUMs (Biologics Drug Ratio and Unified intact Mass analysis), an automated Python pipeline to simplify data analysis of the mean drug-to-antibody ratio and structural integrity analysis of biologics and bioconjugates. A graphical user interface (GUI) and a Web app interface have also been implemented to make it user-friendly for users with little to no prior programming knowledge.

## Graphical abstract

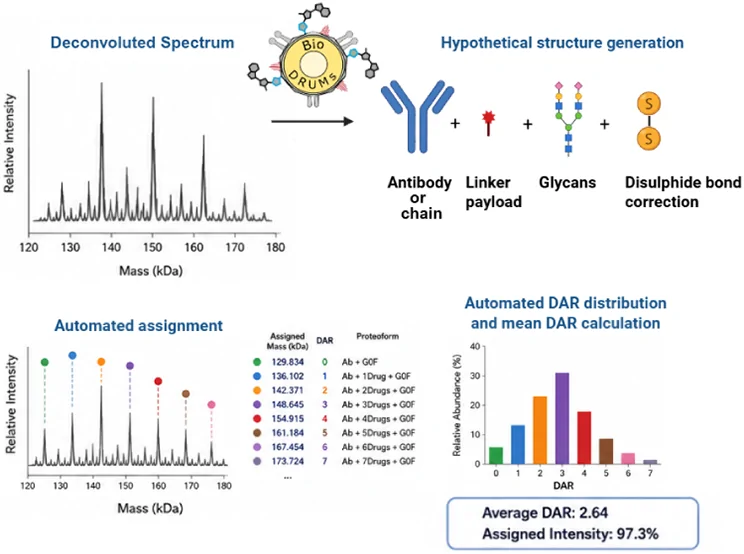

## Introduction

Antibody-drug conjugates (ADCs) are shaping the present age of targeted therapies. These immunoconjugates combine the specificity of monoclonal antibodies (mAbs) to engage disease-associated targets with the pharmacological properties of conjugated drugs to exploit their biological effects on targeted cells.^1^ The conjugation reaction between synthetic drug molecules and a mAb can vary widely, according to the employed chemistry. A mixture of ADC species, characterized by a range of drug-to-antibody ratio (DAR) values, could be present in a single batch.^2^ Liquid chromatography coupled to mass spectrometry (LC-MS) is the technique of choice for providing the required accuracy in determining DAR values.^3^ The currently employed LC-MS methods for DAR determination are mostly performed on high-resolution mass spectrometry (MS) analyzers.^4^ However, they can differ depending on the degree of protein unfolding during analysis (denaturing vs native MS conditions^5,6^ ). The native MS approach is preferred for the analysis of noncovalently bound ADC samples (where interchain disulfide bonds are no longer present after mAb bioconjugation) compared to the analysis performed under denaturing conditions.^7,8^ Nevertheless, it is not always applicable to *in vivo* samples and complex matrices (e.g., animal plasma). On the other hand, reversed-phase liquid chromatography (RPLC) coupled to mass spectrometry has also been used to retrieve *in vivo* mean DAR in pharmacokinetic studies for covalently and noncovalently bound ADCs.^9–11^ Commonly, the DAR value can be indirectly calculated from the ratio of two ADC analyte concentrations, hence the conjugated payload and total antibody. The conjugated payload is usually measured by LC-MS on low-resolution analyzers (e.g., triple quadrupole) by using collision-induced dissociation for absolute quantitation.^12^ Instead, total antibody is typically monitored using ligand-binding assays or LC-MS/MS through signature peptide quantitation.^13^ However, this indirect DAR evaluation is remarkably affected by error propagation from the two individual measurements.^14^ Consequently, intact mass analysis by high-resolution mass spectrometers (HRMS) is essential to retrieve an accurate DAR value in a single analysis. In this approach, the deconvoluted signals are subsequently used to calculate the average DAR value by weighting the intensity of each DAR species.^15^ This can be done both on glycosylated and deglycosylated samples. In the first case, all glycoforms present in the sample should be known or characterized prior to DAR analysis, or they can be inferred from the host expression system.^16^ Despite advances in instruments and sample preparation, one of the most critical steps for *in vivo* mean DAR experiments is data analysis and interpretation. Over the last years, many bioinformatics tools have been developed to deconvolve mass spectra to obtain robust protein masses.^17–19^ These platforms can perform spectral deconvolution and intact-protein mass analysis, automated mass assignment, and, in some cases, ADC-specific DAR calculation. Examples include BioPharma Finder,^20^ BioPharmaView and Biologics Explorer,^21^ MassHunter BioConfirm,^22^ and Byos.^23^ These platforms provide charge-state deconvolution, peak integration, mass-accuracy assessment, proteoform or modification annotation, and batch or template-based processing. However, the outputs, accepted file formats, workflow architecture, and QC terminology differ between platforms. Several tools also have a limited number of proteoforms that can be used to build the ADC deconvoluted mass library and require a commercial license. Some of them require deep knowledge of ADC structure, disulfide bond arrangement, glycan composition, linker payload bioconjugation delta mass impact, that limits their utility for unexperienced users. In addition, routine software outputs are mass accuracy, signal-to-noise ratio, and peak intensity, but most of them do not explicitly check the percentage of deconvoluted signal explained by the user-defined proteoform model or an explicit count of alternative isobaric DAR assignments. This is crucial for thorough sample characterization (number of MS peaks that are explained within a set mass accuracy threshold) and for ensuring that the reported mean DAR is representative and unambiguously reported (in cases where a deconvoluted mass matches species with different DAR values). In this work, we introduce the BioDRUMs (Biologics Drug Ratio & Unified intact Mass analysis) pipeline, which integrates data from HRMS instruments and postprocesses them in an automated manner (Figure 1). BioDRUMs is complementary to commercial deconvolution programs and is designed to standardize downstream interpretation across deconvolution platforms, by simplifying ADC species identification and proteoform building (by giving some default values for IgG-based ADC or the most common bioconjugation strategies) or automated DAR distribution characterization and mean DAR calculation, especially on *in vivo* samples. It is compatible with different table formats coming from different mass deconvolution algorithms. It performs automated deconvoluted mass assignment (both on glycosylated and deglycosylated samples) and mean DAR analysis on *in vivo*/*in vitro* samples and automatically plots the relative percentage of each DAR species over time. Specifically, BioDRUMs provides (i) automated generation of proteoform hypotheses combining antibody masses, DAR ladder, linker degradation species, and glycoforms; (ii) unified calculation of mean DAR and structural integrity (SI) across in vitro and in vivo datasets; (iii) quality-control (QC) metrics (explained area percentage, isobaric species count) for intact MS data; and (iv) a GUI-based workflow and a Web app interface that ingests different deconvolution output formats. To our knowledge, no existing toolchain offers this combination of proteoform modeling, isobaric species handling, DAR/SI quantitation, and QC in a Python package.

**Figure 1:**
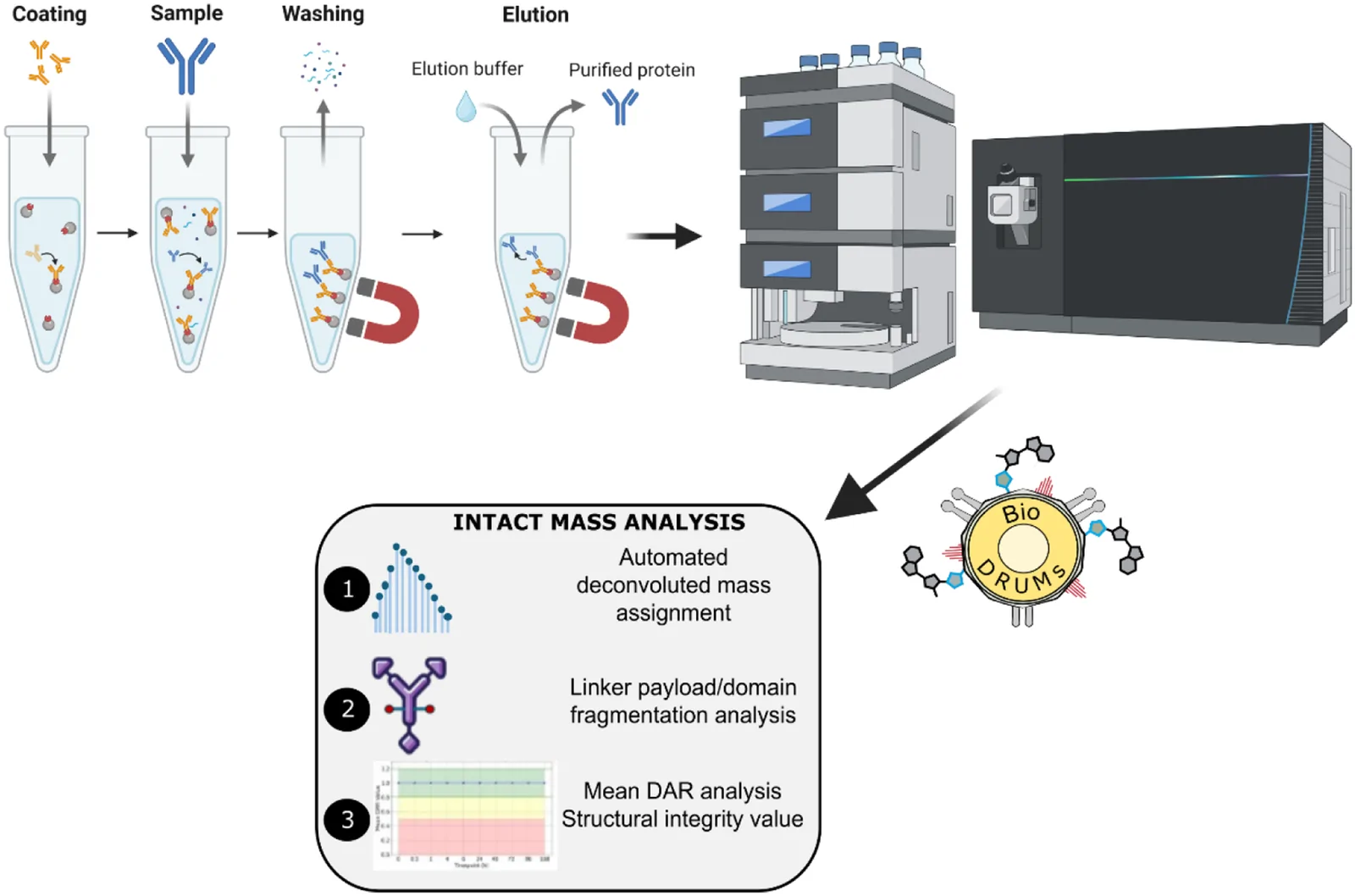
BioDRUMs contribution to the intact mass analysis workflow. After sample preparation steps and LC-MS analysis, BioDRUMs can be used to simplify the data analysis process, both by assigning the mass of the identified proteoform species after signal deconvolution and automating mean DAR analysis and DAR distribution for ADCs and structural integrity for multidomain biotherapeutics/monoclonal antibodies. Created in BioRender. Di Ianni, A. (2026) https://BioRender.com/nqh0zqr.

## Materials and Methods

### Chemicals and Reagents

The valine-citrulline PABC MMAE Trastuzumab ADC, ADC8, and the unconjugated Trastuzumab were produced at Merck KGaA (Darmstadt, Germany). The Biotin SP-conjugated AffiniPure Goat Anti-human IgG Fc fragment-specific capture antibody was purchased at Jackson ImmunoResearch (Product code number: 109-065–098, Cambridge House, United Kingdom). PBS tablets were obtained from Life Technologies (18912014, Thermo Scientific, CA, USA). MS-grade acetonitrile (ACN), formic acid, and MS-grade water were delivered from Sigma-Aldrich (St. Louis, MI, USA). Streptavidin sepharose magnetic beads were purchased from Cytiva (28985738, GE Healthcare, Marlborough, MA), and Smart Block was purchased from Candor Biosciences (113125, Wangen, Germany). Blank mouse plasma used for the preparation of mouse plasma *in vitro* stability samples was obtained from BioIVT (Westbury, NY, USA).

### PK Mouse Study Design

For mouse plasma PK samples, independent technical replicates were not performed because of the limited volume per animal available (approximately 10/20 μL, needed to be shared across multiple bioanalytical assays, e.g., immunoassays for total/conjugated antibody quantitation and complementary ADC characterization such as conjugated payload quantitation). This situation is representative of a typical exploratory in vivo ADC/biologic study, where sample volume is often a limiting factor. Together with real *in vivo* samples, using the same reference ADC/biological standard in mouse plasma, a calibration curve at different concentration levels (commonly from 5 to 100 μg/mL) is normally processed with BioDRUMs as a system suitability test (see Table S1). Across this concentration range, the resulting mean DAR values showed a relative standard deviation (RSD) of 3.56%, indicating that, under similar conditions, single-injection results are expected to be representative.

### Sample Preparation for Middle-Up/Top-Down Analysis

150 μL of streptavidin-coated magnetic beads (20.0 mg/mL, Cytiva) were washed three times in PBS and reconstituted in 430 μL of PBS assay buffer. Then, 70 μL of Biotin S-SP-conjugated AffiniPure Goat anti-human IgG Fc fragment-specific capture antibody (1.3 mg/mL, Jackson Immunoreasearch, Baltimore, USA) was coated on the achieved streptavidin magnetic bead suspension for 2 h on a digital test-tube rotator (Labinco, Breda, Netherlands). Afterward, the coated beads were washed three additional times with PBS and resuspended in a final volume of 1500 μL. 10 μL of ADC/protein mouse plasma-spiked sample was loaded into a 700 μL Protein LoBind 96-well plate from Waters (Milford, USA). The ADC molecules were enriched by pipetting 150 μL of bead suspension per well in a final volume of 500 μL of PBS and incubated on a Thermomixer at room temperature at 1000 rpm. After the enrichment, samples were rinsed twice with PBS and two times with MS-grade water. The deglycosylation step (overnight, pH 7.4, 37 °C) is optional and depends on the molecule (some ADC payloads are sensitive to the pH conditions of sample preparation). Immunoenriched samples were eluted into clean MaxPeak HPS vials (Waters, MA, USA) using 30 μL of 1% FA in water, Smart Block 1:50. Reduction with TCEP (after deglycosylation) can be used to separate the conjugated light and heavy chains.

### Liquid Chromatography–Mass Spectrometry for Intact/Subunit Analysis of ADC Samples

5 μL of ADC-enriched samples were injected in a full-loop configuration into a Dionex UltiMate 3000 LC system coupled to an Orbitrap Eclipse mass spectrometer (Thermo Scientific, Bremen, Germany) on a 5 μL sample loop. The sample was loaded onto a MabPac® RP, 4 μm, 1500 Å pore size, 0.15 × 150 mm column (Thermo Scientific, PN ES907), that was maintained at 55 °C for the analytical separation. The mobile phases consisted of 0.1% v/v HCOOH in water (Phase A) and 0.1% v/v HCOOH in ACN (Phase B). The intact ADC-enriched plasma samples were eluted at a flow rate of 1 μL/min and the following gradient was used: 0.0–6.0 min, 20% B; 6.0–12.0 min, 20–85% B; 12.0–20.0 min, 85% B; 20.0–20.1 min, 85–95% B; 20.1–24 min, 95%B; 24.0–24.1 min, 95–85% B; 24.1–34.0 min, 85% B; 34.0–34.1 min, 85–20%B; 34.1–45.0 min, 20%B. For MS detection in nanoEasy configuration, a full-scan MS spectrum (700–4000 m/z) including 10 microscans was acquired in positive ion mode, without the lock mass option enabled, with an in-source CID of 80 V. The nominal MS resolution was set to 15000 at full width at half-maximum at m/z 200. A target value of 3.00 × 10^6^ ions was selected for the automatic gain control setting with a maximum injection time of 50 ms. The other MS parameters were spray voltage at 2.0 kV, capillary temperature at 275 °C, and S-lens RF level at 80%. For the subunit analysis, ADC-enriched plasma samples were eluted at a flow rate of 1 μL/min, and the following gradient was used: 0.0–4.0 min, 5% B; 4.0–6.0 min, 5–25% B; 6.0–18.0 min, 25–32% B; 18.0–20.0 min, 32–80% B; 20.0–28.0 min, 80%B; 28.0–28.1 min, 80–95%B; 28.1–39.0 min, 95% B; 39.1–50.0 min, 5% B. For MS detection in nanoEasy configuration, a two-segment Full Scan (FS) MS experiment (m/z 266.7–4000) was acquired in positive ion mode, without the lock mass option enabled. Specifically, the first Full Scan segment (0–19 min) for the light chain (LC) MS analysis was set to a resolution of 120,000 at full width at half-maximum (FWHM) at m/z 200, without any in-source fragmentation, for 5 microscans in Profile mode. A target value of 3.00 × 10^6^ ions was selected for the automatic gain control (AGC) setting with a maximum injection time of 200 ms. The spray voltage was set at 2.0 kV, capillary temperature, 275 °C, and S-lens RF level at 60 V. Concerning the second FS segment (19–50 min) for heavy chain (HC) MS analysis, the mass spectrometer resolution was set to 30,000 FWHM at m/z 200 for 10 microscans in Profile mode. A target value of 3.00 × 10^6^ ions was selected for the AGC setting with a maximum injection time of 54 ms. The other MS parameters were spray voltage at 2.0 kV, capillary temperature at 275 °C, and S-lens RF level at 60 V.

### BioPharma Finder Pipeline

MS spectra deconvolution was performed with the Biopharma Finder software 4.0 (Thermo Scientific) using the ReSpect algorithm for isotopically unresolved spectra with sliding windows for heavy chain and intact ADC analysis. Light chain analysis was carried out using the Xtract algorithm for isotopically resolved spectra. The DAR species were searched by putting the linker payload monoisotopic and average masses as variable modification in the Modification Editor window. The input mass range was set from 1400 to 4000 m/z. The output mass range was set to 10–200 kDa, with a minimum required adjacent charge states set to 6, whereas the retention time (R_T_) range was restricted to reduce the computation time, with an R_T_ tolerance of 1 min from the expected average R_T_ of the intact ADC molecule/subunit. The output from BioPharma Finder can be used as a starting template (or by following simple instructions in Table S2) to postprocess the metadata using the unified_DAR_analysis.py module of BioDRUMs.

### BioDRUMs Pipeline

The deconvoluted mass finder.py BioDRUMs module is used to automatically perform ADC/protein structure hypotheses to identify them after protein MS signal deconvolution (see Results and Discussion paragraph “BioDRUMs Python Modules” for details).

The unified DAR analysis.py BioDRUMs module enables three types of analysis:

1) Mean DAR/SI analysis over time/concentration using the following equation (eq 1):

1 $$mean\;DAR/SI=\frac{\overset{n}{\underset{i=0}{\sum }}\;area\;intensity\;DAR/SI^{ith}xnominal\;DAR/SI\;value}{total\;\mathrm{antibody}\;area\;intensity}$$

**Equation 1**. Mean DAR/SI Calculation. For structural integrity, an analogous metric is computed by assigning SI = 1 to intact full-length species and SI = 0 to nonintact species. An example of mean DAR calculation can be found in the Supporting Information.

All PTMs on the antibody backbone preserve the integrity of the ADC/biologic structure, and then, by default, an SI value of 1 is assigned to them. Moreover, unknown masses or unassigned degradation products can be incorporated into SI calculations; in this case, an SI value of 0 is assigned. SI should not be confused with the percentage of efficacious molecules but provides an overall estimate of the percentage of the intact structure (unmodified or with potential PTMs) that could potentially exert the pharmacological effect.

2) Explained area percentage (%) analysis, namely, the percentage of identified MS deconvoluted peaks from the total number of MS peaks. Commonly, an acceptable threshold to ensure good sample characterization is set to 75%. This percentage could drop when approaching the limit of detection of the instrument, particularly for *in vivo* samples.

3) DAR species characterization: Once identified, the individual assigned species are plotted as the relative intensity percentage to the total intensity of all identified species for each timepoint/concentration (default threshold is a 4% relative percentage of the most intense MS deconvoluted peak), according to the set mass accuracy threshold (default mass accuracy is set to 30 ppm). Then the identified species are univocally described by a species ID number that is mapped into an Excel sheet report (named as “Species_ID_Mapping”).

The Python pipeline is freely accessible at https://github.com/adiianni8/BioDRUMs. A Web app interface (using the Streamlit package) has also been developed and is freely accessible from a link in the BioDRUMs GitHub page with recommended search parameters.

## Results and Discussion

### BioDRUMs Python Modules

BioDRUMs are based on two Python modules. The first module has been called Deconvoluted mass finder.py and is used to facilitate ADC structural hypotheses and their identification after mass spectrometry signal deconvolution (Figure 2). It automatically includes ADC linker payload mass shift after bioconjugation with the antibody backbone (in cases where enzymatic/chemical bioconjugation is performed; see Table S1) for transglutaminase and interchain/engineered cysteines. Other mass shifts can be set by the user according to their own bioconjugation technology. Additionally, degradation forms of linker payload can potentially be included (especially suitable for *in vivo* characterization, Figure 2A). Moreover, if deglycosylation cannot be performed for ADC instability or antibody-conjugated payload sensitivity to sample preparation conditions, BioDRUMs can incorporate different glycosylation patterns in the deconvoluted mass calculation (both on the intact molecule or on the single chains,^24^ Figure 2B). The second module is called unified DAR analysis.py and automatically performs three analyses: mean DAR analysis over time/concentration, explained area percentage, and DAR species analysis. It can be interfaced with both commercial and open-access deconvolution software for intact protein characterization, and it only needs a template Excel file to perform all analyses (Table S2). In principle, the output of any table-formatted deconvolution data analysis software for intact mass analysis is suitable for BioDRUMs as long as it contains a column with the DAR value of an individual species, a protein name, and a sum intensity column. Data filtering is based on mass accuracy thresholds of identified species set as 30 ppm and a relative abundance of 4% related to the MS base peak intensity (the most abundant deconvoluted MS peak identified in a sample), but the user can modify these thresholds according to their needs. Both modules take less than 1 min to complete their tasks on a standard laptop (16.0 GB RAM, Windows 10–11/64-bit operating system).

**Figure 2:**
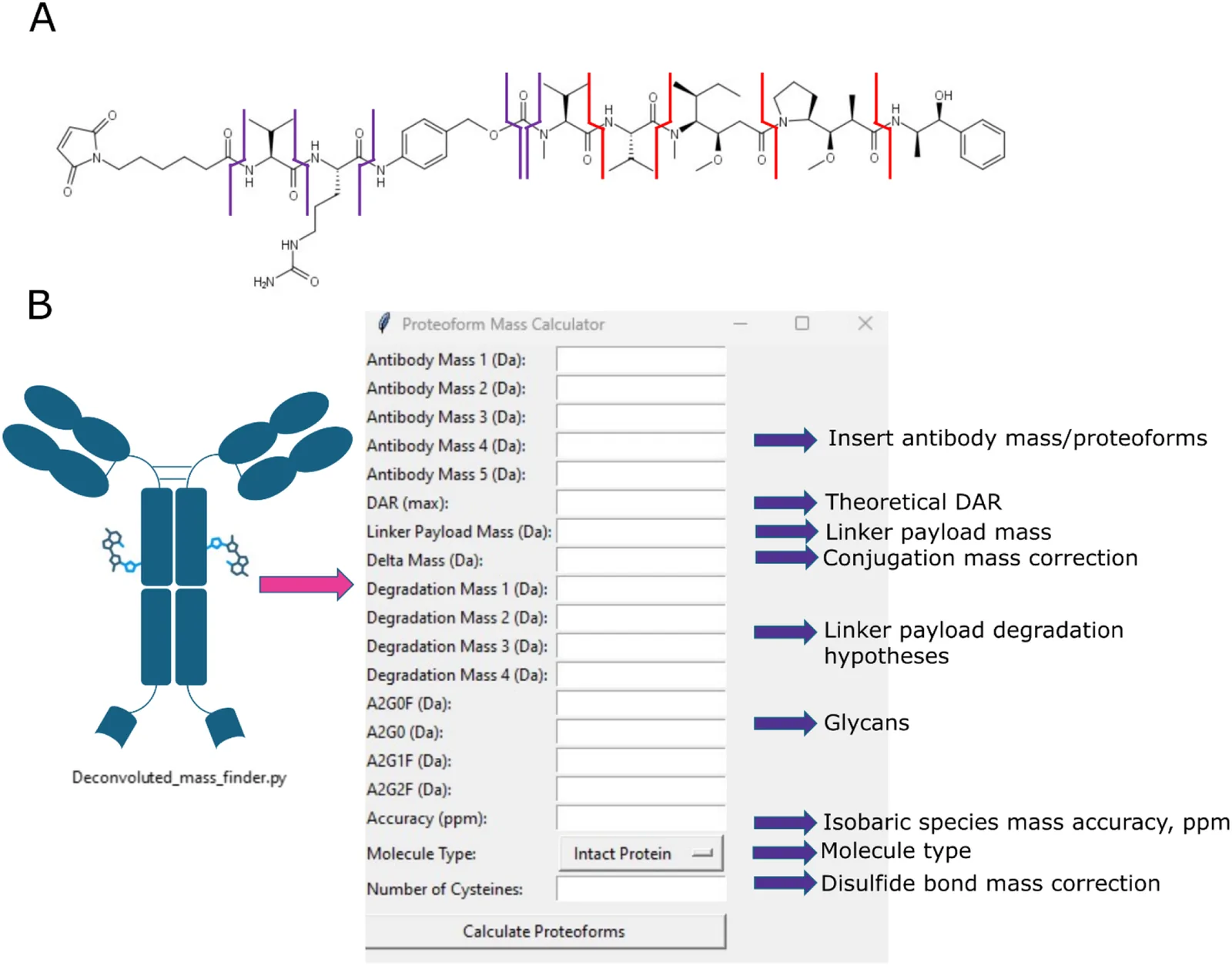
Proteoform mass calculation GUI workflow. (A) Example of plausible degradation forms for maleimido-caproyl-valine-citrulline para-amino benzoyl carbamate (PABC) monomethyl Auristatin E. In purple, hydrolytic cleavages from plasma carboxypeptidases are highlighted. Potential catabolic hot spots on the payload are shown in red. (B) Deconvoluted mass finder graphical user interface: different fields need to be compiled to generate all potential structure hypotheses for deconvoluted mass identification from the theoretical DAR to DAR0 (Table S1).

### Mass Identification and Structure Hypothesis Generation

The first step after LC-MS intact protein/subunit analysis by mass spectrometry is spectral deconvolution, which converts m/z peaks’ envelope from multicharged proteins into masses. At this stage, having information on the molecular weight of a protein or protein conjugate is necessary for targeted identification of the main unmodified protein/ADC species. For high-resolution analyses, isotopic resolution can be achieved for every charge state from masses of ∼30 kDa or smaller on the Orbitrap mass analyzer. Then, reducing the disulfide bonds of the antibody could lead to monoisotopic mass determination on the conjugated light chain only. On the heavy chain, this would simplify potential isobaric species combinations, but information on structural integrity could not be directly assessed from samples. BioDRUMs automatically identifies potential proteoforms and generates different DAR species (from a specified theoretical DAR value to DAR0), using a dedicated GUI to simplify its usage. It automatically corrects the mass of the protein (or protein chain if working on a subunit level) for the number of paired cysteines (involved in a disulfide bond or in the conjugation with linker payload) present in the sequence. This is extremely useful when additional cysteines are incorporated into the protein sequence for site-specific conjugation or mutations of the hinge region to stabilize IgG4 scaffolds. It will also compute all combinations of intact linker payload and its potential degradation forms using all deglycosylated proteoforms, but also on the main glycosylated proteoforms (for antibody-based candidates). Eventually, it will also count the number of potential isobaric species for each DAR level (within the provided mass accuracy in the GUI) for both glycosylated and deglycosylated proteoforms and give a complete Excel list table.

### Mean DAR/Structural Integrity Value Determination and Identification of Structural Liabilities

Once the generated proteoforms are identified and assigned with good mass accuracy from the experimental deconvoluted masses, the mean DAR value is automatically calculated by the unified DAR analysis.py BioDRUMs module (see details in the methods section) and plotted according to an independent variable (e.g., timepoint for pharmacokinetic or *in vitro* stability studies, concentration for method qualification or applicability assessment). This is still achieved via a GUI (Figure 3), where the user needs to upload the compiled template Excel output file (Table S2). Optionally, the user can define the name of the molecule, the *x*-axis plot variable, the study ID and type, and matrix information to be included in the final plots.

**Figure 3:**
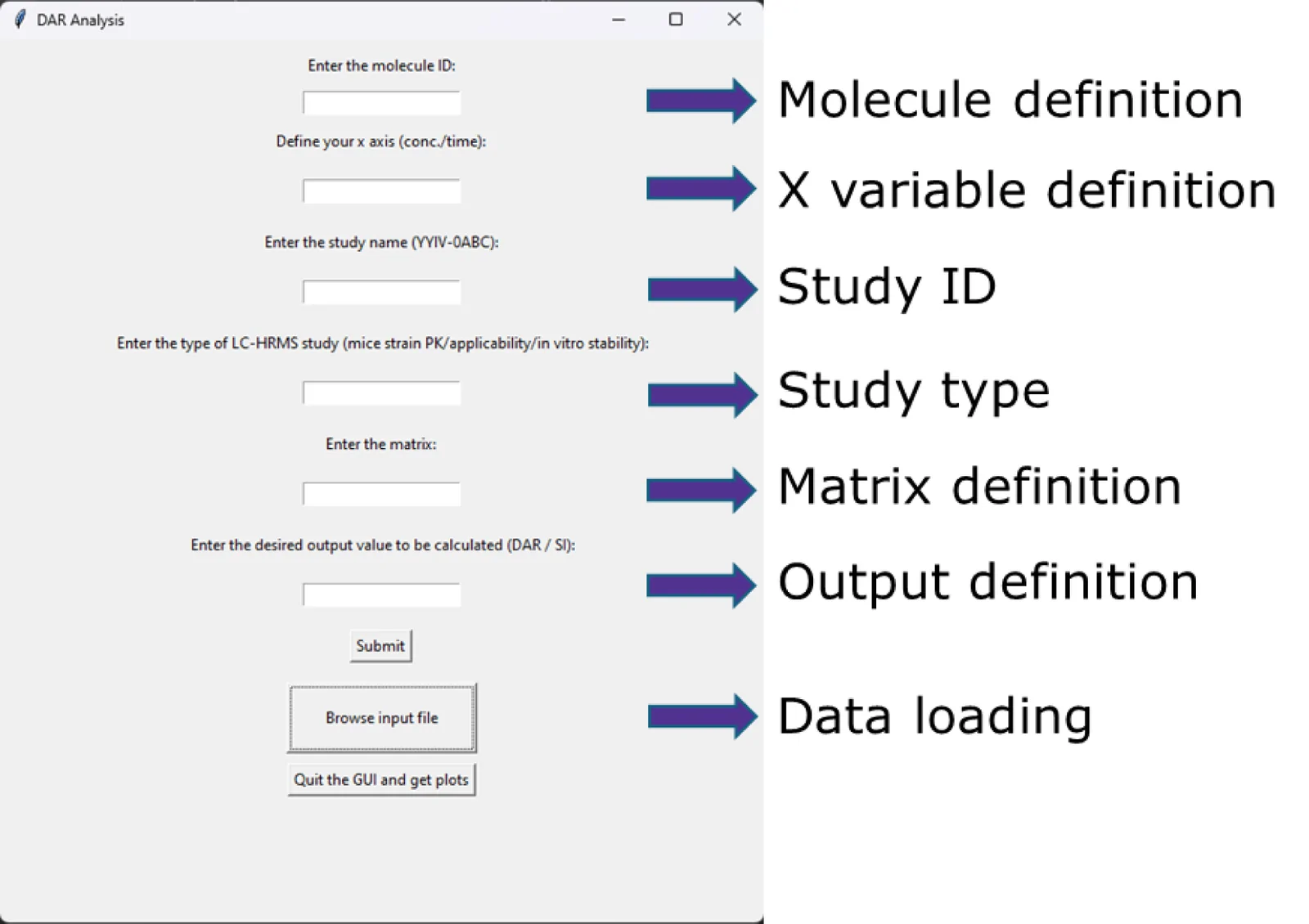
DAR analysis GUI workflow. The user can define the molecule ID, type of study, matrix type, and the output DAR (for protein/peptide conjugates and subunits) or structural integrity (SI) value (for unconjugated antibody/bispecifics/multispecifics). There is also a button to enable the upload of metadata (via an Excel spreadsheet).

### Use Case One: Mean DAR Value Determination for a Trastuzumab ADC in an *In Vitro* Stability Study

To illustrate a real case scenario, we analyzed data from an *in vitro* mouse plasma stability (0–168 h) of a valine-citrulline para-aminobenzoyl carbamate (PABC) monomethyl auristatin E (MMAE)-conjugated trastuzumab using the BioDRUMs pipeline. The bioconjugation was made by using a maleimide-reactive linker on an engineered cysteine in the CH3 domain of the heavy chain. Thus, a maximum of two conjugation sites are amenable to linker payload conjugation (then a maximum DAR2). Data were generated on a Tribrid Orbitrap Eclipse operating in nano-mode configuration (see the methods). Samples were deglycosylated overnight using PNGase-F, to simplify the assignment of different ADC species and reduce the number of potential isobaric identifications in the samples (Figure 4A,B).

**Figure 4:**
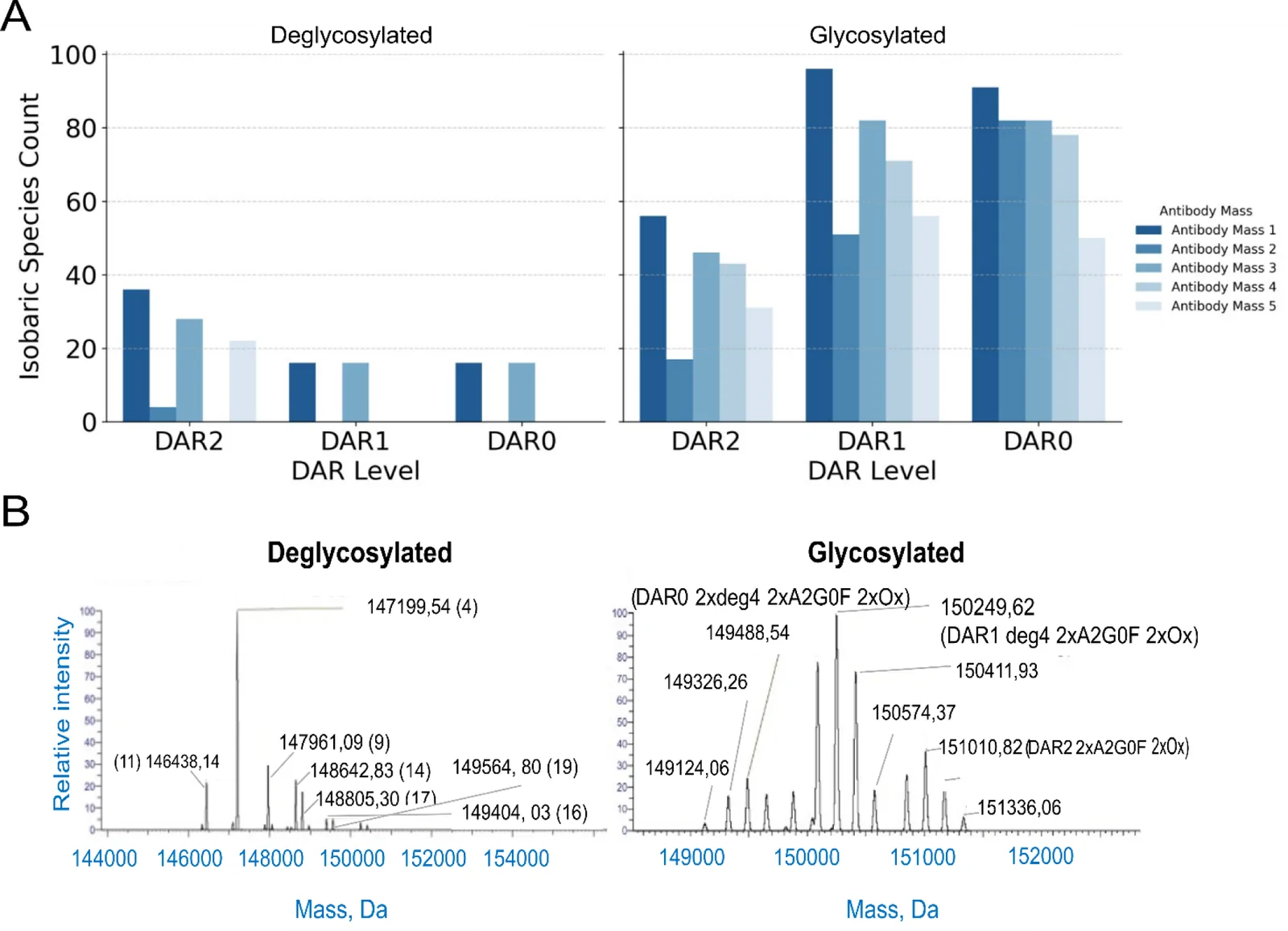
Impact of deglycosylation on the number of potential isobaric species for Trastuzumab ADC. (A) Deconvoluted mass finder module in BioDRUMs can predict the number of potential isobaric species, given the antibody masses, the intact linker payload and its degradations, and their glycans (for glycosylated samples) for all proteoforms and associated DAR levels. (B) Deconvoluted mass spectrum of a deglycosylated Trastuzumab ADC spiked in mouse plasma compared to a glycosylated ADC sample. Main DAR species and glycoforms are highlighted for the glycosylated Trastuzumab ADC. The other peaks are shifted by ∼162 Da, which corresponds to one galactose residue from the main DAR species. Deglycosylated Trastuzumab ADC species can be found in Table S3. In this case, due to the high number of predicted isobaric species in glycosylated samples, a deglycosylation step was performed to reduce potential bias/misassignments in mean DAR calculation (Table S4).

In cases where several proteoform hypotheses are isobaric within the chosen mass tolerance, BioDRUMs does not attempt to mathematically resolve their individual contributions; instead, it flags the number of possible isobaric assignments per DAR level. This provides a qualitative indication of assignment ambiguity, which users should interpret in the context of prior knowledge (e.g., expected glycoform repertoire) and, where possible, confirm with orthogonal experiments such as deglycosylation, subunit analysis, or targeted MS/MS.^14^ Moreover, BioDRUMs isobaric species output could suggest whether, for the specified ADC structure and DAR, a deglycosylation or a reduction is needed to lower the theoretical number of isobaric species eventually found in real samples. Commonly, by working at the subunit level or applying N-deglycosylation by PNGase F (as in this case, Figure 4B), it is enough to reduce potential ambiguity in unique DAR species assignment. 22 total isobaric species were found considering the experimentally obtained deconvoluted masses in the glycosylated trastuzumab ADC plasma sample (Figure 4B, Table S4), based on the built ADC library in BioDRUMs. Conversely, no isobaric deglycosylated ADC species were found on deglycosylated trastuzumab ADC samples (Figure 4B, Table S4).

Results of mean DAR analysis are shown in Figure 5. The deconvoluted mass spectra of Trastuzumab ADC at two representative timepoints with all automated assignments by BioDRUMs are shown in Figure 5A. Mean DAR remained stable over all *in vitro* stability timepoints, settling within 20% of DAR loss compared to t0 (green area, Figure 5B). This suggests good stability of the valine-citrulline PABC MMAE linker payload, justifying its use in the first clinically approved ADCs on the market.^25^ Furthermore, the percentage of identified MS deconvoluted peaks from the total number of MS peaks was above the 75% threshold for all of the samples, denoting an in-depth characterization of the main ADC species present in each individual sample (Figure 5C). A total of 21 ADC species were identified in the time course of the *in vitro* stability (Figure 5D). The main identified species is a DAR 1 (species 4) Trastuzumab proteoform with lysine-clipping modification on both the heavy chains C-terminus, followed by species 9 (unmodified DAR 2 Trastuzumab ADC with no glycosylation) and 11 (unconjugated Trastuzumab with two deg4 linker payload modifications; see Tables S1 and S3). Moreover, the BioDRUMs isobaric-species output did not identify competing isobaric assignments for the experimentally observed ADC species across the analyzed time points (Tables S3 and S4), increasing confidence in the calculated mean DAR values.

**Figure 5:**
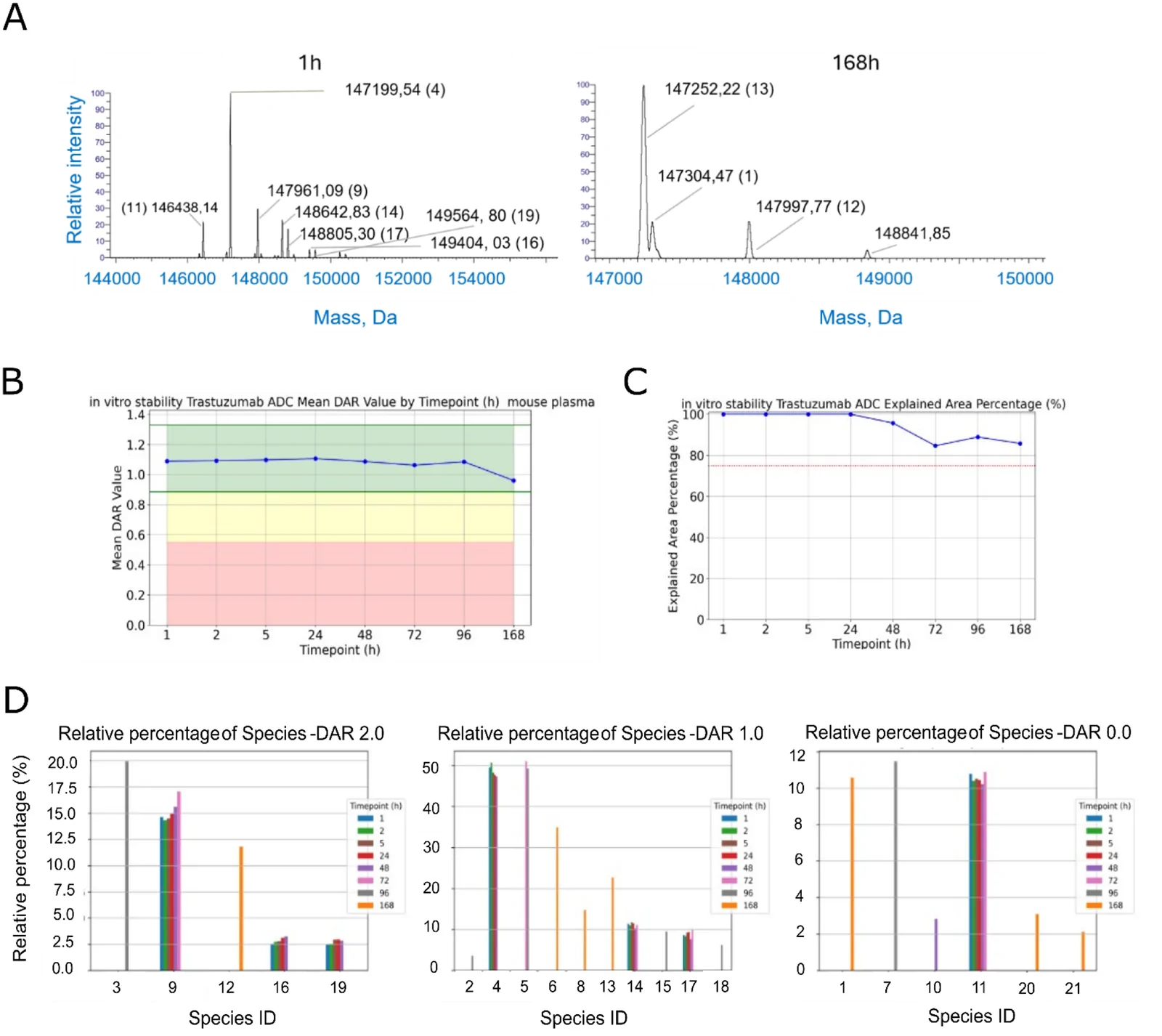
BioDRUMs output for Trastuzumab ADC in vitro mouse plasma stability. (A) Deconvoluted mass spectra of two representative in vitro stability timepoints (1 and 168 h), showing the different Trastuzumab ADC DAR species identified by Deconvoluted mass finder.py. (B) Mean DAR analysis over time in mouse plasma stability samples; (C) explained area percentage (%) analysis for each timepoint; (D) DAR species characterization over time. Relative percentages of different ADC DAR species were plotted for each timepoint. An Excel spreadsheet with the species ID mapping is also generated from the unified DAR analysis.py BioDRUMs module.

### Use Case Two: Mean Structural Integrity Value and Glycosylation Profiling for Unconjugated Trastuzumab in a Pharmacokinetic Study

To demonstrate the handling of structural integrity of a general biologic candidate, pharmacokinetic (PK) samples for the unconjugated trastuzumab mAb were taken to perform the calculation of a structural integrity (SI) value. In this specific case, no linker payload degradation pattern needs to be defined, but the loss of chains *in vivo* and the glycosylation pattern should be investigated. However, modifications on the protein backbone such as N-terminus pyroglutamylation, cysteine and methionine oxidation, protein cysteinylation, and glutathionylation were considered. It is up to the user to decide how PTMs can affect the overall SI, but so far, from our experience, no loss of activity is driven by PTMs on the antibody backbone, and then, they are normally deemed as nonimpacting SI proteoforms. However, these proteoform variants differ in their biological properties such as effector functions (for example, antibody-dependent cellular cytotoxicity), complement activation, and pharmacokinetic properties (e.g., serum half-life).^26,27^ Moreover, the main glycosylated proteoforms present in the sample were included in the proteoform hypothesis generation (Figure 6A). A summary of the results is shown in Figure 6. No trastuzumab fragments due to domain losses were observed, hence, the SI of this candidate was not affected *in vivo* (Figure 6B). The main identified species is the intact trastuzumab with A2G0F and A2G1F glycans, with glycine and lysine C-clipping (species 5) proteoform, followed by species 11 (with 2 A2G1F glycans with glycine and lysine C-clipping) and species 10 (with A2G0F and A2G1F glycans, with two glycine and lysine C-clippings, Figure 6C,D). Conceptually, SI values close to 1 indicate that the deconvoluted signal is largely dominated by intact full-domain species, whereas lower SI values would reflect an increasing contribution of chain loss or antibody fragments.

**Figure 6:**
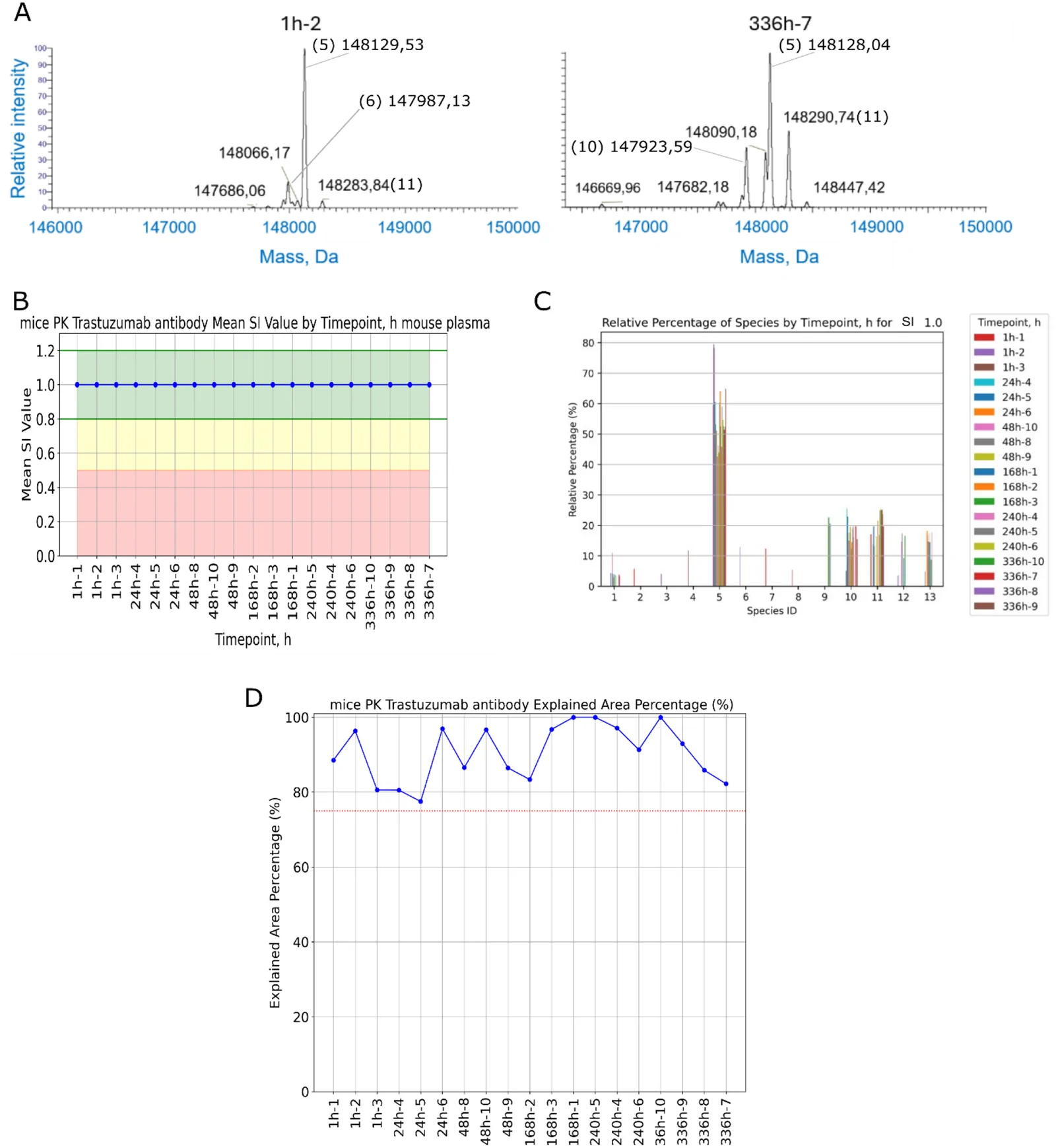
BioDRUMs output for unconjugated Trastuzumab antibody in vivo pharmacokinetic plasma sample analysis. (A) Deconvoluted mass spectrum of two representative PK timepoints (1 and 336 h), showing the different Trastuzumab glycoforms identified by Deconvoluted mass finder.py. (B) Mean SI analysis over time in in vivo pharmacokinetic samples; (C) proteoform characterization over time. The relative percentages of different glycosylated antibody species were plotted for each timepoint. An Excel spreadsheet with the species ID mapping is also generated from the unified DAR analysis.py BioDRUMs module. (D) Explained area percentage (%) analysis for each timepoint.

### Use Case Three: Subunit Mean DAR Analysis

Another example of the broad applicability of BioDRUMs in ADC bioanalysis is the characterization of ADC-conjugated subunits. This is normally achieved by reducing the interchain disulfide bonds of the antibody, releasing the conjugated heavy and light chains. In this case, the BioDRUMs pipeline was carried out on a DAR 8 IgG-based ADC, here named ADC8. Here, the bioconjugation site is on interchain cysteines, after reduction of the disulfide bonds, giving rise to a potential maximum DAR of 8. By analyzing it at the subunit level, this will result in the conjugated light chain with up to one linker payload (LC DAR 1 as maximum DAR) and the conjugated heavy chain with up to three linker payloads (HC DAR 3 as maximum DAR). A summary of the results is shown in Figure 7. In Figure 7A, a representative spectrum for conjugated LC (left panel) and HC (right panel) is shown at 0 h and 168 h of the in vitro mouse plasma stability. From Figure 7B, the BioDRUMs mean DAR analysis output is shown. Briefly, no major issues in the mean DAR stability over time were seen in plasma samples. The LC resulted in a stable DAR of 1 over the plasma stability time course (Figure 7B). The HC showed a slight decrease in mean DAR from 3.0 to 2.7, especially at 96 h and 168 h (Figure 7C). However, both conjugated chains had a DAR loss of less than 20% from t0 (green zone in the mean DAR analysis plot). From the BioDRUMs DAR species characterization output, the unmodified species LC DAR 1 (species 1), which represents the main species at 0 h, turned into LC DAR1 with one hydrolysis (+18.015 Da, species 2) and LC DAR 1 with one hydrolysis and one oxidation (species 9, Figure 7D). Instead, as far as it concerns the HC, the HC DAR 3 with one lysine clipping (species 20), which is the main species at 0 h, turned into HC DAR 3 with one hydrolysis and lysine clipping (species 17). Species 22 and 21 represent the associated species of HC DAR 3 and HC DAR 3 with one hydrolysis but still carrying one A2G0F glycan (for incomplete deglycosylation, Figure 7E). From this BioDRUMs output, information about the evolution of the different ADC species over time in *in vitro* and *in vivo* samples can be retrieved.

**Figure 7:**
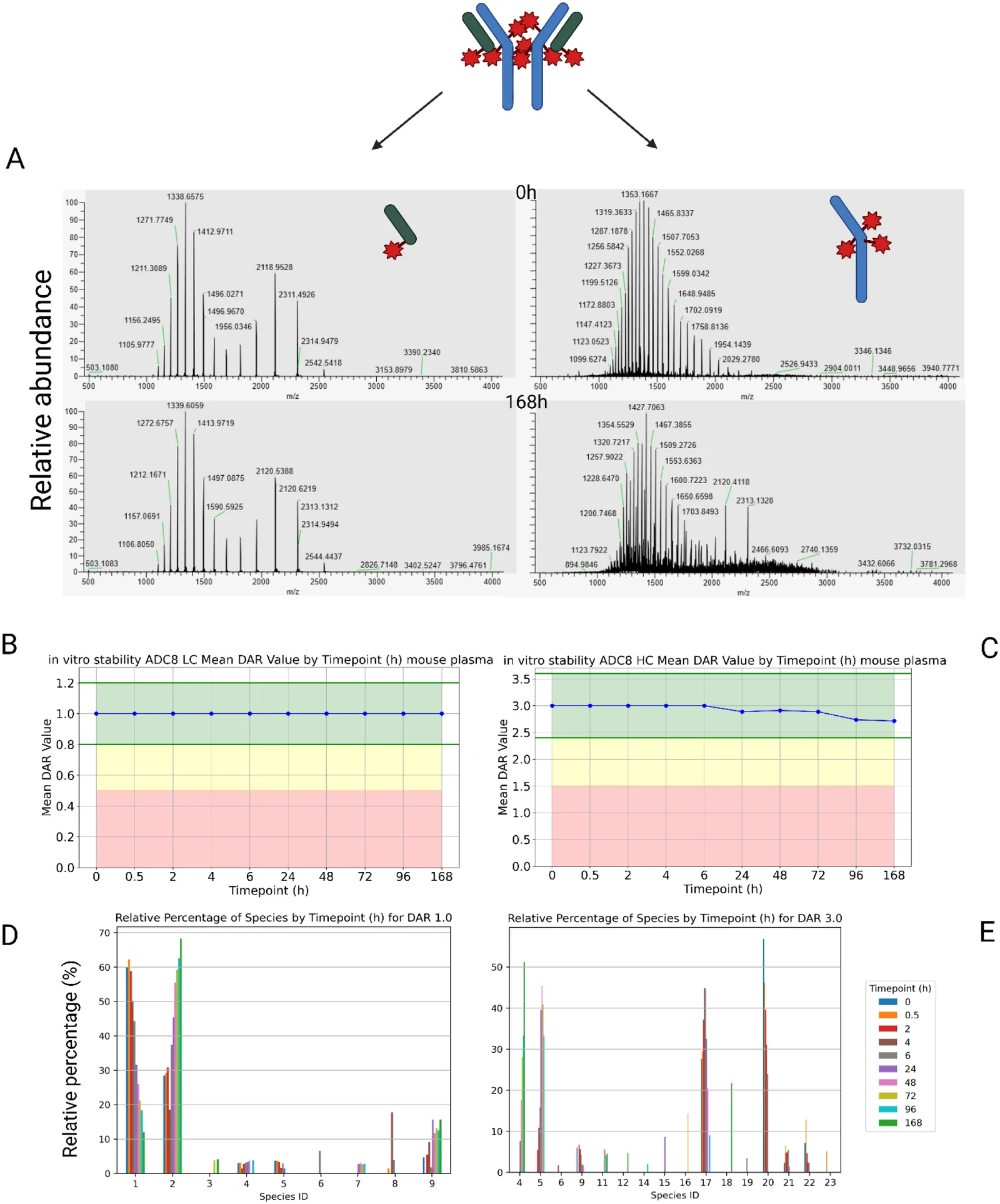
BioDRUMs output for the conjugated subunit analysis of ADC8 from in vitro plasma stability samples. (A) MS spectra of conjugated LC and HC at 0 h and 168 h in mouse plasma stability samples. Mean DAR analysis over time in mouse plasma stability samples for B) LC and C) HC. DAR species characterization over time for D) LC and E) HC for different timepoints. Other HC DAR species (from DAR 2 to DAR 0) are shown in the Supplementary Information (Figure S1). Created in BioRender. Di Ianni, A. (2026) https://BioRender.com/lwafzhu.

## Conclusions

While replicate injections are desirable for robust estimation of precision, they are frequently impractical in small-animal studies due to limited sample volumes and the need to support multiple analytical end points. Under these realistic constraints, the main routes to improving confidence in mean DAR and structural integrity values are (i) maximizing the data quality within a single injection and (ii) standardizing and automating data processing. To address data-processing variability, we described BioDRUMs, a flexible, user-friendly Python package for the analysis of various ADC/biologics data sets. BioDRUMs provides features for data quality control (explained area percentage and isobaric species count), data analysis on glycosylated/deglycosylated intact proteins/subunits, and data plotting and interpretation with an easy-to-use package. We included three examples to illustrate the utility of the different BioDRUMs functions for the analysis of intact protein conjugates/biologics and subunit analysis datasets and the user manual on how to compile the template and the GUI in Tables S1–S3. The three use cases presented here showed the versatility of BioDRUMs for in vitro ADC stability analysis, in vivo PK structural integrity assessment of an unconjugated antibody, and subunit-level DAR characterization for ADC8. The pipeline is particularly suited for low-volume in vivo studies, where replicate measurements are not feasible, and standardizing single-injection analysis is essential. A Web app interface has also been developed, enabling users to run BioDRUMs without a local Python setup or package installation.

## Supplementary Material















## Notes

Figure S1: HC DAR species characterization for ADC8 (DAR 2 and DAR 0) (PDF)
Table S1: Exemplary GUI compilation of Deconvoluted mass finder.py: generic Trastuzumab IgG1-based ADC conjugated via maleimido-vc-PABC-MMAE (XLSX)
Table S2: Exemplary GUI compilation of unified_DAR_analysis.py and Excel DAR sheet template (XLSX)
Table S3: Summarized mean DAR results output (XLSX)
Table S4: Trastuzumab ADC proteoforms output (XLSX)
Table S5: Species ID mapping in vitro stability ADC8 LC mouse plasma (XLSX)
Table S6: Species ID mapping in vitro stability ADC8 HC mouse plasma (XLSX)
