# Supplementary material for: BioDRUMs: An Open-Source Python Pipeline for Automated Mean Drug-to-Antibody Ratio and Biologics Structural Integrity Analysis from High-Resolution Mass Spectrometry Data
Source: J Am Soc Mass Spectrom. 2026 Oct 5;37(11):2861–72. doi: 10.1021/jasms.6c00289 (PMC13741909; doi:10.1021/jasms.6c00289)
Supplement: Supplementary file 1 [file js6c00289_si_001.pdf]

Supporting Information

BioDRUMs: an open-source Python pipeline for automated mean drug-to-antibody ratio and biologics structural integrity analysis from high-resolution mass spectrometry data

Andrea Di Ianni[1,2], Francesco Molinaro[1], Luca M. Barbero[1], Kyra Cowan[3], Federico Riccardi Sirtori[1]*

AUTHOR ADDRESS

[1]NBE-DMPK Innovative BioAnalytics, Merck Serono RBM S.p.A., an affiliate of Merck KGaA, Darmstadt, Germany, Via Ribes 1, 10010 Colleretto Giacosa (TO), Italy.

[2]University of Turin, Molecular Biotechnology Center, Department of Molecular Biotechnology and Health Sciences, University of Turin, 10126 Turin, Italy

[3]New Biological Entities, Drug Metabolism and Pharmacokinetics (NBE-DMPK), Research and Development, Merck KGaA, Frankfurterstrasse 250, 64293 Darmstadt, Germany

*Correspondence to andrea.di-ianni@merckgroup.com

KEYWORDS. Drug-to-antibody ratio; data analysis automation; antibody-drug conjugates; biologics; intact mass analysis; biotherapeutics development

Supplemental Figures

Figure S1- HC DAR species characterization for ADC8 (DAR 2 and DAR 0)

Supplemental Tables

Table S1- Exemplary GUI compilation of Deconvoluted mass finder.py: a generic Trastuzumab IgG1-based ADC conjugated via maleimido-vc-PABC-MMAE (.xlsx)

Table S2- Exemplary GUI compilation of unified_DAR_analysis.py and Excel DAR sheet template (.xlsx)

Table S3- Summary mean DAR results output (.xlsx)

Table S4- Trastuzumab_ADC_Proteoforms_Output_Table S4.xlsx

Table S5- Species_ID_Mapping_ in vitro stability ADC8 LC_mouse plasma.xlsx

Table S6- Species_ID_Mapping_ in vitro stability ADC8 HC_mouse plasma.xlsx

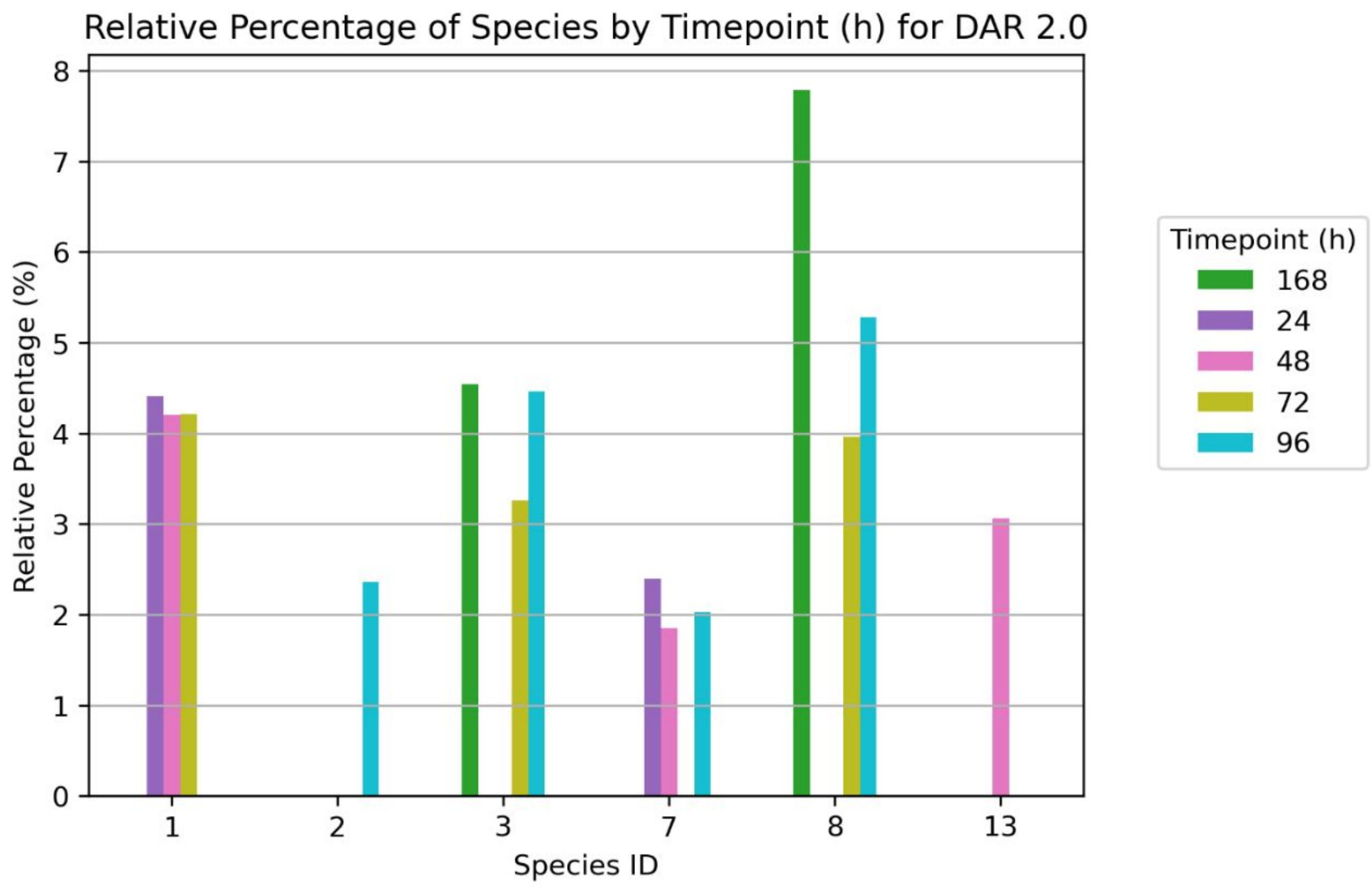

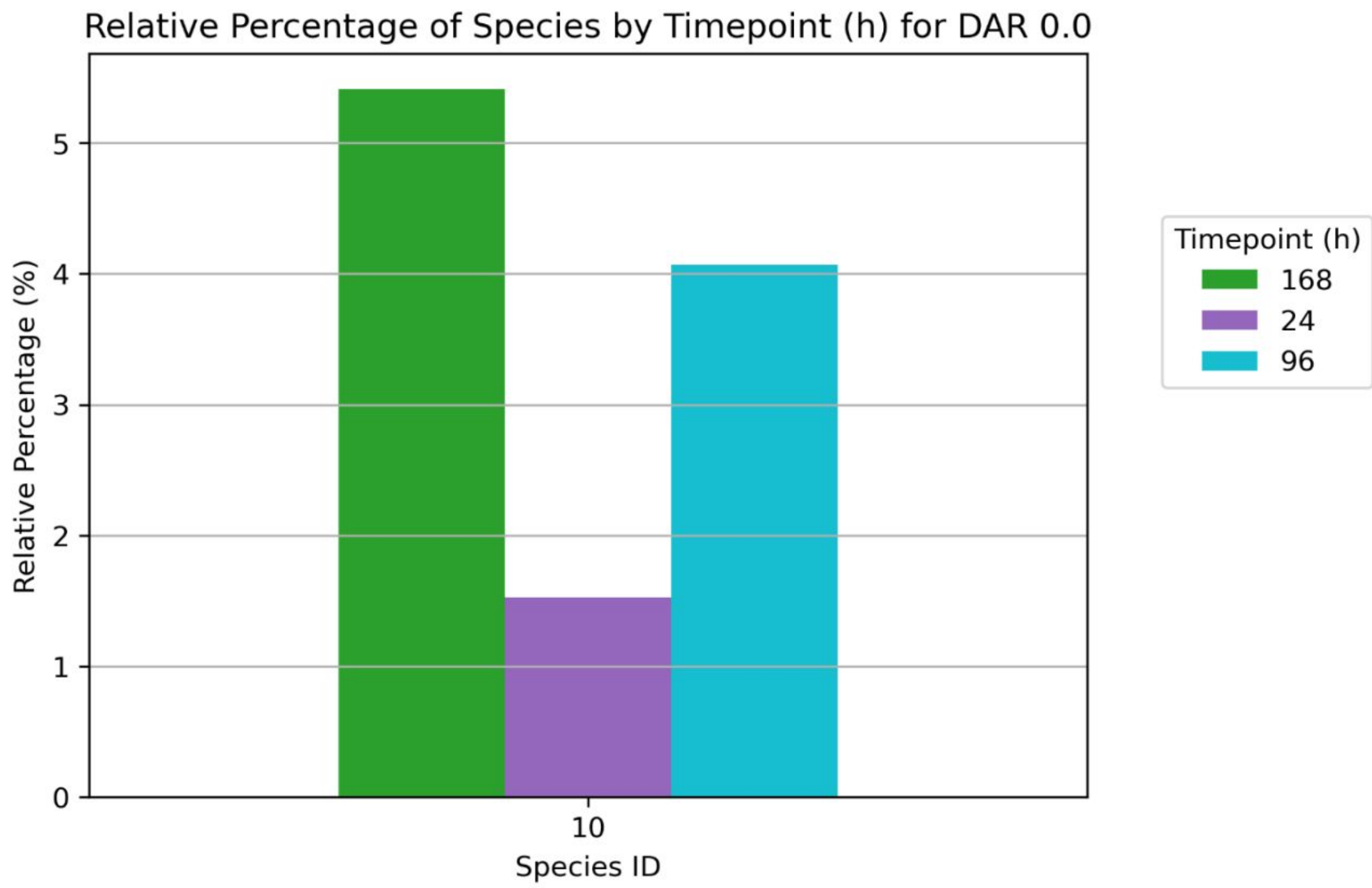

Figure S1- HC DAR species characterization for ADC8 (DAR 2 and DAR 0). Species ID can be found in Table S6.

Following Equation 1 in the main manuscript we provide practical values to calculate mean DAR for Trastuzumab ADC (e.g., at 1h in mouse plasma)

$$mean\ DAR/SI = \frac{(2*7.19*10^{8}+1*2.55*10^{9}+0*3.95*10^{8})}{3.66*10^{9}} = 1.09$$
